# The Role of Anthracyclines in Cardio-Oncology: Oxidative Stress, Inflammation, and Autophagy

**DOI:** 10.1155/2022/9862524

**Published:** 2022-04-14

**Authors:** Leonardo Schirone, Stefano Toldo, Eleonora Cianflone, Valentina Sala, Ernesto Greco

**Affiliations:** ^1^“Sapienza” ‒ University of Rome, Rome, Italy; ^2^Virginia Commonwealth University, Richmond, USA; ^3^Magna Graecia University, Catanzaro, Italy; ^4^University of Turin, Turin, Italy

In past decades, much effort was put to define the molecular mechanisms underlying the development of anthracycline-induced cardiomyopathy. As a result, administration protocols were refined and dosages were lowered according to new guidelines, whenever it was possible. To manage the delicate balance between tumor eradication and cardiac health of the patients, an increasing number of multidisciplinary cardio-oncology wards were created in past years in several hospitals. This new synergistic discipline is aimed at transferring from the bench to the bedside the novel approaches that come from basic and translational research.

Among the anthracycline, doxorubicin (DOX) is one of the most studied due to its wide use as a chemotherapeutic agent in a number of malignancies and due to the frequent development of adverse side effects. These include doxorubicin-induced cardiomyopathy (DCM), a potentially lethal condition that may manifest both acutely or chronically and fail to respond to current therapies used to treat cardiovascular disease [1]. Acute cardiotoxicity occurs within 2-3 days from the administration of the drug and results in the development of myopericarditis, palpitations due to tachycardia, electrocardiographic changes (e.g., nonspecific ST-T alterations), and premature atrial and ventricular beats [2]. Rarely, acute left ventricular (LV) failure may develop. Despite the fact that the mechanisms for these alterations are not clear, several lines of evidence focused on a role for doxorubicin-induced myocardial edema, which is reversible and may be treated [1]. The incidence of acute DCM is approximately 11%, while chronic DCM is estimated at 1.7% of all treated patients [1]. The lack of accurate epidemiological data is mainly due to the extensive time range (from 30 days to more than 10 years) in which chronic DCM may emerge from the moment of doxorubicin administration. Moreover, the incidence varies largely depending on the dose that is used, varying from 1.7% (below 500 mg/m^2^) to 36% (over 600 mg/m^2^) [3]. Lastly, being very young or old and having a history of cardiovascular disease are risk factors for congestive heart failure (CHF), a condition that has a very poor prognosis (50% of the affected patients die within 1 year from congestive heart failure) [4].

The development of neglected side effects in a tissue with very low (and controversial) regenerative properties like the myocardium has always induced scientists to find therapeutic targets to achieve cardioprotection without altering the antineoplastic activity of doxorubicin. The toxicity exerted on proliferating cells is probably based on completely different mechanisms compared than those affecting cardiomyocytes. Curiously, dozens of possible mechanisms have been proposed in past decades and can be clustered in oxidative stress-based, gene expression-based, and cell death-based.

Anthracyclines strongly affect mitochondrial respiration and integrity in cardiomyocytes, favoring the reactive oxygen species (ROS) production. In this special issue, Doroshow and colleagues studied the effect of different chemotherapeutic drugs on cyanide-resistant oxygen consumption and ROS production in an *in vitro* model of cultured adult primary rat cardiomyocytes. Apart from 5-iminodaunorubicin and mitoxantrone, all the tested drugs increased the oxygen consumption of cardiomyocytes. Considering that a ROS scavenging treatment with catalase or acetylated cytochrome c reduced oxygen consumption and that the author detected increased hydrogen peroxide production in response to DOX, it is likely that the mechanisms underlying the cardiotoxicity of all the hereby tested anticancer quinones include ROS overproduction [5].

In this special issue, Carrasco et al. reviewed the latest findings on the role of oxidative stress-mediated molecular mechanisms underlying the development of doxorubicin-induced cardiomyopathy. Oxidative stress is the first and most widely accepted mechanism of doxorubicin cardiotoxicity. Doxorubicin can be reduced to an unstable semiquinone metabolite that targets the cardiomyocytes due to its high affinity to cardiolipin, a phospholipid whose density is exceptionally high in mitochondria, which are impressively numerous in cardiomyocytes, due to their high energy demand [6]. In mitochondria, the molecule cycles and produces free radicals that damage these organelles, facilitated by the fact that antioxidant enzymes and molecules are poorly expressed in cardiomyocytes [7]. Damage to mitochondria may in turn lead to permeability issues to the mitochondrial membranes impairing the electron transport chain, causing a positive feedback that further increases the production of ROS and promotes apoptosis [8].

DOX is also known to interfere with the three isoforms of the nitric oxide synthase (NOS) enzyme, which catalyze the formation of NO from L-arginine and O_2_, as studied by Wang et al. (see below).

Besides, many studies in past decades focused on the ability of anthracyclines to chelate free iron, forming a complex that reacts with molecular oxygen and triggers ROS production [9]. However, the iron-DOX complex is nowadays considered to have only a minor role in the pathology of DCM, while free iron accumulation in the myocardium has been proven to trigger apoptosis through mechanisms that are independent of oxidative stress [10].

In their work, Carrasco et al. critically review the different mechanisms defined in past decades and cluster most of them as oxidative stress-related [8]. The authors highlight that many of the cardiovascular risk factors associated with anthracycline-induced cardiomyopathy correlate with increased susceptibility to oxidative stress. Coherently, most of the current preventive and mitigative pharmacological strategies that target the development of this life-threatening condition positively affect the patient's redox status. These include statins, ACE inhibitors, beta-blockers (carvedilol, nebivolol) and polyunsaturated fatty acids. The authors underline that the past trials aimed at directly scavenging ROS likely failed because of their simplistic design, compared to the novel approaches that counteract ROS by stimulating the native antioxidant response, e.g., activating the long-lasting Keap1/Nrf2/ARE pathway. Moreover, genetic variability may account for the interindividual different susceptibility to develop anthracycline-induced cardiomyopathy, as detailed by Yang et al. in this special issue (see below).

Following this line of evidence, the work from Zhang et al. provides new insight into the ROS-scavenging approach to mitigate the effects of DOX administration. In this study, mice were treated for 10 days (5 days before and 5 days after acute DOX treatment) with O-methylated flavone oroxylin A (OA), which is known to be beneficial against inflammation and cancer. OA administration prevented the DOX-associated myocardial atrophy, systolic derangements, and cell death. The authors linked the protective effects exerted by OA administration to preserved levels of expression of sirtuin 1 (Sirt1), a critical deacetylase that is downregulated by DOX. They reported that OA is not protective in cardiac-restricted Sirt1 KO mice, showing that OA relies on Sirt1 activity to prevent acute DOX-induced cardiotoxicity [11].

A different approach was used by Wang et al, who used electroacupuncture (EA) at Neiguan acupoint (PC6) to prevent the development of DOX-induced cardiomyopathy in iNOS-deficient and cardiac-specific arginase 2- (Arg2-) deficient mice. In this study, the authors found that DOX stimulates the production of nitrogen monoxide (NO) and that its levels correlate with iNOS upregulation and Arg2 downregulation. However, EA at PC6 impaired DOX-induced NO upregulation and exerted protective cardiac effects in treated mice, by reducing cardiac dysfunction and hypertrophy. These functional benefits were mechanistically linked to the activity of iNOS and Arg2: iNOS-deficient mice displayed a better heart function than the wild type after DOX treatment but EA did not further improve their phenotype; conversely, cardiac-specific Arg2-KO mice developed a worse heart function and did not benefit from EA. Together, these findings demonstrate that EA at PC6 may represent a novel approach for alleviating DOX-induced cardiomyopathy by preventing NO overproduction [12].

Besides oxidative stress, Yang et al. reviewed the genetic variability that has been associated with increased risk of developing DCM, which involves mutations in genes implicated in metabolism, autophagy, ROS scavenging, mitochondrial function, DNA damage, endoplasmic reticulum stress, inflammation, and apoptosis. These include CYBA, GSTA1, NCF4, RAC2, ABCC1, ABCC2, CAT, UVRAG, GCN2, TCL1A, TLRS, C282Y, Hmox1, CBRs, MYH7, TNNT2, and TTNtv. This work brings together the findings of different genetic clinical studies and, despite studies from larger cohorts being still needed, represents a useful starting point for future research projects on the subject. Defining genomic combinations of polymorphisms that predict an increased risk of developing anthracycline-induced cardiomyopathy may represent a cornerstone to guide clinicians to personalized rational drug use and, eventually, to the use of combined cardioprotective strategies.

Nowadays, much attention is being paid to avoid reaching a critical life-long dose of anthracycline. However, at the state of the art, clinicians are often cornered between the urgency of treating aggressive cancers and the risk of causing iatrogenic heart disease. This is particularly true for those patients that received chemotherapy in pediatric age and that face a second oncologic disease in their adulthood. In the future, merging traditional ROS scavenging approaches, the new mechanistic molecular insights beneath anthracycline-induced cardiomyopathy and genetic screening will hopefully result in personalized therapies that will help vulnerable patients to be safely cured of cancer.

*Leonardo Schirone* *Stefano Toldo* *Eleonora Cianflone* *Valentina Sala* *Ernesto Greco*
